# Guidelines for Enhanced Recovery After Trauma and Intensive Care (ERATIC): Enhanced Recovery After Surgery (ERAS) Society and International Association of Trauma Surgery and Intensive Care (IATSIC) Recommendations: Paper 1: Initial Care—Pre and Intraoperative Care Until ICU, Including Non‐Operative Management

**DOI:** 10.1002/wjs.70002

**Published:** 2025-07-22

**Authors:** Timothy C. Hardcastle, Christine Gaarder, Zsolt Balogh, Scott D'amours, Kimberly A. Davis, Amit Gupta, Shahin Mohseni, Paal A. Naess, Shanisa Naidoo, Tarek Razek, Simon Robertson, Hayaki Uchino, David Zonies, Jade Whing, Michael J. Scott

**Affiliations:** ^1^ Inkosi Albert Luthuli Central Hospital Durban South Africa; ^2^ Department of Surgical Sciences University of KwaZulu‐Natal Durban South Africa; ^3^ Oslo University Hospital Ulleval and University of Oslo Oslo Norway; ^4^ John Hunter Hospital and University of Newcastle Newcastle Australia; ^5^ Liverpool Hospital and University of Sydney Sydney Australia; ^6^ Division of General Surgery Trauma and Surgical Critical Care Yale School of Medicine New Haven Connecticut USA; ^7^ JPN Apex Trauma Centre All India Institute of Medical Sciences New Delhi India; ^8^ Department of Surgery New Al Jahra Hospital Al Jahra Kuwait; ^9^ School of Medical Sciences Orebro University Orebro Sweden; ^10^ Montreal University Hospital and McGill University Montreal Canada; ^11^ Canberra Hospital Canberra Australia; ^12^ Department of Surgery University of Washington Seattle Washington USA; ^13^ Norfolk and Norwich University Hospital Norwich UK; ^14^ Department of Anesthesiology and Perioperative Medicine University of Rochester Rochester New York USA; ^15^ Leonard Davis Institute for Health Economics University of Pennsylvania Philadelphia Pennsylvania USA; ^16^ University College London London UK

**Keywords:** enhanced recovery after surgery, ERAS, major trauma, perioperative care, polytrauma

## Abstract

**Background:**

Enhanced recovery after surgery (ERAS) protocols reduce length of stay, complications, and costs for elective surgical procedures. It remains challenging to implement ERAS concepts in the acute trauma patient due to deranged physiological reserve from the penetrating or blunt trauma producing altered physiology. However, systems of care improve access to early intervention and potentially reduce mortality. These consensus guidelines examine optimal pre‐hospital, resuscitation‐room, intra‐, and post‐operative treatment, systems of ethical management, and overall care for trauma patients in the post‐resuscitation phase of care. The guideline is presented in three parts, this being part 1.

**Methods:**

Experts in aspects of management of trauma surgical patients and intensive care were invited to contribute by the International ERAS Society and IATSIC. PubMed, Cochrane, Embase, and MEDLINE database searches on English language publications were performed for ERAS elements using the patient intervention comparator outcome (PICO) consensus questions created by the expert group. Studies were selected with particular attention to randomized clinical trials, systematic reviews, meta‐analyses, and large cohort studies, reviewed, and summarized recommendations were graded using the grading of recommendations, assessment, development and evaluation (GRADE) system. These recommendations based on current best evidence, with extrapolation from elective patient studies, where appropriate, were followed by a modified two‐round Delphi method to validate final recommendations. Several ERAS components are already standard of care within national and society guidelines and are endorsed. The bulk of the text focuses on key areas pertaining specifically to trauma care of major trauma and polytrauma in the ICU‐requiring group.

**Results:**

Overall 37 aspects of trauma care were considered with multiple PICO questions and sub‐points. Consensus was reached after two rounds of a modified Delphi process involving all authors, with minor adjustments to some phrasing required, but with 87% overall agreement on all statements (100% agreement on 31 of the main statement sets, prior to minor edits to address the points of difference for the rest with 100% total agreement thereafter). None were rejected outright. The recommendations and level of evidence for each aspect of trauma care that may impact on improved recovery and reduced length of hospital stay are presented with grade of recommendation.

**Conclusions:**

The guidelines relating to initial care and decision‐making are presented in part 1 of the Guidelines. These guidelines are based on current best evidence for an ERAS approach to patients who have had major injuries and polytrauma. The guidelines are not exhaustive but collate the best available evidence on important components of care for this patient population. As some of the evidence is extrapolated from elective surgery and non‐trauma emergency surgery, some of the components need further evaluation in future studies.

## 1 Introduction

### 1.1 Background

Enhanced recovery after surgery (ERAS) protocols reduce length of stay, complications, and costs for elective surgical procedures. The ERAS Society has published many guidelines on elective surgery and more recently emergency laparotomy [1, 2, 3]. To date trauma has largely been excluded from such guidelines, along with surgical intensive care patients, because these patient populations do not generally allow for the usual ERAS principles to be applied to their care. This is because trauma, caused by kinetic energy transfer to the body, whether through blunt or penetrating forces, is multisystem and potentially multi‐regional and results in extreme surgical stress prior to contact with healthcare providers which is what the ERAS guidelines have aimed to prevent or reduce in the elective environment. Although the guidelines relating to emergency laparotomy began to address the emergency cohort they did not include emergency *trauma* laparotomy hence an additional argument for a new guideline.

There is no comprehensive current guideline on how to adapt ERAS pathways to torso and polytrauma patients who require admission to intensive care (ICU), both for post‐operative and non‐operative care and for all age groups throughout their hospital stay. The ability to apply many of the ERAS guidelines may be problematic as there is a need to address reduced mobility, requirement for ICU mechanical support, inability to apply early removal of devices, and need for variable nutritional support as well as a number of other controversial questions within the care of the injured. The potential benefits include following evidence‐based structured approaches to care, proactive interventions, and reduction of adverse events leading to reduced morbidity and mortality after trauma.

The principles behind the ERAS concept include pre‐operative, intra‐operative, and post‐operative guidelines of care aimed at reducing physiological stress, thereby reducing the hospital length of stay, complications, and to ensure early discharge [4]. The challenge is that most of the pre‐operative aspects (counseling, carbohydrate loading, antibiotic and thromboprophylaxis, limiting premedication, and fasting) are simply not possible in the resuscitation environment of the unplanned trauma patient. The intra‐operative aspects (avoiding drains, judicious fluids, analgesia concepts, and normothermia) are at odds with the (patho)physiological response to injury and interventions. The routine application of the post‐operative ERAS guidelines remain challenging due to the common occurrence of ileus, and acute kidney injury which mostly necessitates the maintenance of urinary catheter or nasogastric tubes. Routine early enteral feeding may not be feasible during the prescribed timeframe because of the discontinuity of the gastrointestinal tract up to several days postinjury. Using non‐steroidal and epidural analgesia is often precluded by residual coagulopathy and acute kidney injury.

A trauma‐specific guideline that addresses all aspects of the ‘polytrauma’ patient, irrespective of whether due to penetrating or blunt trauma, with major tissue injury, traumatic shock, systemic inflammatory response, and at risk of organ failure after torso and extremity injury (while excluding burns, drowning and isolated brain injury), is thus required to guide safe and ideal practice [5, 6]. To bridge this gap, the International Association of Trauma Surgery and Intensive Care (IATSIC) approached ERAS to undertake a collaborative guideline development process for the application of modified early recovery and best‐practice steps in the management of the acute trauma patient, both pre‐, peri‐, and post‐operatively and during recovery, firstly in ICU and also in the ward prior to discharge. This also includes non‐operatively managed patients.

In addition to the challenge of a lack of guidelines comes the extreme variation in the ability of health systems to manage major trauma, despite the criteria for trauma centers and systems evolving over the last four decades [7, 8, 9, 10, 11, 12, 13]. Lower‐ and middle‐income regions face huge trauma burdens with significant resource challenges while higher income countries often face a reducing operative experience because of higher proportions of blunt trauma lending itself to a more non‐operative management (NOM), along with a reduction in open surgical experience. Furthermore, many surgeons are largely devolved from ICU patient management, an essential aspect of care for the polytrauma patient [14, 15, 16]. To date there are only three published studies of ERAS concepts applied to trauma care, and these demonstrated the feasibility of selected aspects and challenges with other aspects but included only patients not requiring ICU admission, or prolonged ventilation [17, 18, 19].

For the sake of readability and grouping of recommendations the results of the ERATIC guidelines have been divided into three papers with part 1 covering the introduction and main methods, the PICO questions related to initial management, operative and non‐operative care, part 2 covers the post‐operative and intensive care aspects (with some overlap from part 1 highlighted at the end of part 1), and part 3 covers the aspects of ethical decision‐making, end‐of‐life care and trauma systems requirements impacting ultimate outcome (The papers documenting paper 2 and 3 are found as https://doi.org/10.1002/wjs.70004 and https://doi.org/10.1002/wjs.70003 in the *World Journal of Surgery*).

### 1.2 Methods

The project was initiated by the IATSIC group (www.iatsic.org), during the ISW 2022 meeting in Vienna Austria, and a project proposal was submitted to the ERAS Society for approval. Approval was provided and the guidelines development group (GDG) was constituted. The group was from varied surgical (trauma surgeons, general surgeons, vascular surgeons, pediatric, and orthopedic surgeons) or anesthesia critical‐care backgrounds, from various world‐regions and country income levels as well as representing both sexes. Topic lists were generated, and search questions were derived using the patient, intervention, comparator (where available), outcome (PICO), or similar approaches and using the definitions provided in paper 1. The topics were divided across the spectrum to smaller teams of two or three reviewers, with different backgrounds and from different countries, based on their expertise, to perform a literature review of English language publications and then to generate summary recommendations using the GRADE structure, with the grading of the summarized evidence rather than each article considered for inclusion, given this is not a systematic review. All included articles are referenced in the main text summary of the evidence prior to the take‐home ‘Summary of Evidence and the Recommendation’. The time period chosen was 2008–2023 (15 years), but allowing for the inclusion of older sentinel publications where relevant. PubMed, Cochrane, Embase, and MEDLINE database searches on English language publications were performed. Greater emphasis was placed on recent publications, randomized controlled trials, systematic reviews, meta‐analyses and large cohort studies where these existed, however, retrospective or older studies were considered where no other higher level of evidence was available, and if there was particular relevance to either trauma care or trauma intensive care. Where no good evidence was available, expert opinion, if published, was considered, or generated by the group experts. Excluded were letters to editors and small case series. An example flow‐chart of the search process is provided in Addendum 1 (Supporting Information S1) and examples of some of the search strings in Addendum 2 (Supporting Information S2).

Where relevant, prior ERAS guidelines that apply to the PICO questions were also incorporated, or the reader is referred to the topic in those previous guidelines. Expert guidelines from other related organizations in the field of trauma were reviewed, where available and relevant aspects included. Excluded from this review are *isolated* traumatic brain injury, drowning, and burn trauma, unless of relevance to overall patient care. Pediatric‐specific considerations are addressed where deemed relevant in each section and in the ‘systems’ section (Paper 3). The results were then summarized into sections with a summative recommendation graded by level of evidence and grade of recommendation. These 37 recommendations were split into sub‐points and were subject to a two‐round Delphi process with 80% agreement required to accept the recommendation.

## 2 Definitions

In these ERATIC guidelines, trauma refers to the medical and surgical consequences and sequelae of sudden acute kinetic energy transfer to the body causing injury, both intentional and unintentional. The torso constitutes the regions of the body from the neck to the hips including the pelvis, thorax, and abdomen with the typical junctional zones (thoracic inlet, thoraco‐abdominal region, and pelvic outlet). The term major trauma (also called multiple or multi‐system injury) relates to injury to more than one organ system of moderate or severe extent, whereas the term ‘polytrauma patient’ relates to one with multisystem injury *with physiological aberrations* [5, 6].

The pre‐operative phase relates to the time from incident, emergency medical care, initial assessment in the emergency department, and decisions around whether operative or non‐operative management (NOM) are appropriate. The operative phase includes urgent surgical intervention, and this may be in the form of early total care or damage control surgery.

Post‐operative ICU care relates to the admission of patients with major trauma either post‐intervention (surgical or endovascular) or directly from the emergency department in cases selected for NOM, where the patient needs intubation or non‐invasive ventilation, vasoactive drug therapy, or renal replacement therapy.

### 2.1 Commentary

The components of the ERAS emergency laparotomy pathway were reviewed in relation to the patient sustaining acute trauma [1, 2, 3]. However, the physiologic impact varies with the type of trauma and associated blood loss so this warrants a specific trauma pathway (ERATIC). A high level of intraoperative and postoperative resuscitation and monitoring is needed to ensure desired physiological parameters are attained and maintained. Post‐operative intensive care or a high level of care is warranted for this group of patients in the postoperative phase of the pathway.

## 3 Results: Evidence and Recommendations

Regarding the Delphi process there were minor wording changes to six recommendations at the Delphi round 1 and full agreement at round 2. There were no conceptual or major content disagreements noted during either round of the Delphi.

A summary of the ERAS elements for intra‐ and post‐operative care and grading of recommendations with their respective level of evidence are listed in Table 1.

**TABLE 1:** Summary evidence statements and grading.

Question number	PICO question	Recommendation	Level of evidence	Grade of evidence	Delphi agreement
1	In the major trauma and polytrauma patient, what are the pre‐hospital interventions that improve survival and reduce hospital length of stay compared to no intervention?	Rapid transfer to appropriate level of care with minimal fluid therapy or transfusions and attempts at bleeding control are the mainstay of prehospital trauma care. Tranexamic acid may be administered as per local policies. Advanced airway interventions should be restricted to selected patients and long‐distance transfers.	Moderate	Strong	100%
2	In polytrauma patients after initial assessment in the emergency department, what is the decision‐making philosophy the surgeon should follow to improve outcome?	It is recommended that the most experienced (trauma‐trained) surgeon should be the decision‐maker and perform the surgery.	Moderate	Strong	100%
3a1	In trauma patients who meet the current indications for NOM in solid organ injuries when is it still necessary to operate?	There is a high level of evidence to support NOM in hemodynamically normal patients with solid organ injuries after blunt trauma and no other indication for laparotomy provided the patient is appropriately monitored for deterioration.	High	Strong	100%
3a2	What is the role of NOM in penetrating trauma?	For penetrating injuries there is low level of evidence for the use of NOM in isolated liver or kidney injuries. SNOM in penetrating trauma is an option in selected experienced high‐volume centers with rapid access to operation facilities in case of SNOM failure.	Low	Weak	100%
3b	What is the role of repeat imaging after NOM for solid organ injuries in trauma patients?	There is moderate to strong evidence for not repeating CT imaging for uncomplicated solid organ injuries. However, for grade 3–5 splenic injuries that have not been embolized we recommend contrast enhanced US or CT on day 3–5 to exclude pseudoaneurysm.	Moderate	Strong	80%
3c	Does early versus late feeding improve outcome in the trauma patient with solid organ injury?	We strongly recommend early enteral feeding for patients with NOM unless there is a specific contraindication due to the low incidence of harm.	High	Strong	100%
3d	In solid organ injury managed by NOM, what is the role of bedrest compared to normal activity of daily living?	Routine bedrest after NOM in solid organ injuries should be avoided and a limited period of activity restriction is recommended according to the patient age, type (grade) of injury, and organ involved.	Moderate	Strong	100%
3e	In trauma patients who successfully undergo NOM of solid organ injury, what is the safe return to normal activity and sports activity timeline?	Prolonged activity restrictions may be beneficial for contact sports and intense activity.	Moderate	Strong	100%
4a	What are the indications for operative rib fixation and the optimal timing in polytrauma and ICU‐requiring trauma patients with or without TBI?	There is moderate quality evidence to support rib‐fixation for flail chest or > 3 contiguous fractures, those with TBI and in the older patient, to reduce pneumonia, tracheostomy, ICU, and total hospital LOS. There is no evidence to prefer early versus later fixation in selected patient populations.	Moderate	Weak	100%
4b1	What are the indications for chest drains?	Insertion of chest tube: There is strong evidence to support the therapeutic and prophylactic use of chest tubes for drainage of blood and air in the pleural space on an individualized basis.	High	Strong	100%
4b2	What are the management aspects of chest drains in the trauma patient regarding size of tube and placement?	4b2.1 Size and position of insertion of chest tube: The optimal drain position is apico‐posterior. There appear to be no advantage of using larger tubes than 16Fr.	Moderate	Strong	100%
		4b2.2 Complications are common and well detailed. The use of ultrasound can help guide the operator to place the tube appropriately.			
		4b2.3 Smaller tubes and pigtails along with valve‐based mobile drains improve mobility with equivalent drainage ability.			
4c	At what point in the management in the trauma patient with a chest tube is safe removal possible? Removal of chest tube	After appropriate clinical and sometimes radiological assessment, drains are often placed on low pressure suction and transitioned to water‐seal (off suction). Removal is considered when less than 200 mL of fluid drains in 24 h and there is no re‐accumulation of pneumothorax on ‘water‐seal’ in about 6 h. Drains can safely be removed, at either maximal inspiration or expiration, with the patient still in the ICU on positive‐pressure ventilation. Removal should include skin closure with either sutures or occlusive dressing or even cyanoacrylate glue.	Moderate	Strong	80%
5a	In trauma patients with minimal blunt aortic injury (BAI) is operative or non‐operative management required and what is the optimal management strategy compared to open surgery?	There is moderate quality evidence to recommend non‐operative management with medical therapy for minimal aortic injuries, defined as grade I or II by the Society of Vascular Surgery Grading Scale. Serial cross‐sectional imaging is recommended to assure radiographic resolution of injury.	Moderate	Strong	100%
5b	For moderate to severe aortic injury what is the optimal timing of repair: immediate versus delayed?	5b1 There is weak quality evidence to recommend that patients with BAI should undergo TEVAR at, or greater than, 24 h (compared to under 24 h) to improve overall survival.	Low	Weak	100%
		5b2 Open surgery is restricted to a limited subset of patients in extremis.			
6a	For major splenic injury, is main artery versus selective embolization (SAE) better and is it safer to embolize versus observe in high grade injuries?	Spleen injury can be managed non‐operatively for low grade injury and with addition of SAE for higher grade injury. Where to embolize anatomically is controversial, but we conditionally recommend non‐selective SAE.	Moderate	Strong	100%
6b	How effective is immune function after SAE for major splenic injury?	Immune function appears to be retained and immunization is not required.	Moderate	Strong	100%
7a	Which trauma patients benefit from liver packing versus resection?	The hemodynamically compromised patient who does not respond to resuscitation still needs an operation which should be urgently undertaken. With damage control resuscitation strategies, these liver injuries can mostly be managed with systematic packing and TAC until normalized physiology.	Moderate	Strong	100%
7b	For liver injury when is it best to embolize versus observe?	Patients with blunt liver injury and who are hemodynamically stable after resuscitation should undergo NOM. Angiography should be reserved for patients with clinical signs of ongoing bleeding (not only persistent CT‐contrast extravasation or hemobilia).	Moderate	Strong	100%
7c	What is the role of ERCP in the management of hepatic trauma and the optimal timing of drainage for liver trauma related biliary leakage?	For patients with suspected bile duct injury there is moderate evidence supporting closed suction drainage for bile leak. ERCP should be reserved for patients with suspicion of CBD injury and if persistent high output bile leak after several weeks.	Moderate: for closed suction	Weak	90%
			Low: for ERCP		
8a	When should a patient with pancreas injury undergo observation versus operative management?	Grade 1 and 2 pancreatic injuries (no ductal injury) should initially be treated non‐operatively.	Moderate	Strong	100%
8b	When is distal pancreatectomy the procedure of choice?	Grade 3 injuries (to the left of the SMA) can be treated with distal pancreatectomy but there remains the risk of fistula. For those managed with NOM there is a risk of sepsis and pseudocyst.	High	Strong	80%
8c	What is the optimal management of grade 4 and 5 injury?	Grade 4 and 5 injuries should initially be treated with wide drainage rather than primary resection, with subsequent intervention planning as indicated.	Moderate	Weak	90%
9a	In the patient with high grade renal injury—what is the role of NOM with or without angiography? What is the role of NOM versus nephrectomy?	Renal Injury: There is strong evidence supporting NOM in all grade RI in hemodynamically normal or normalized patients (including children) with no other indication for laparotomy. The patients with high grade RI who require an operation will likely undergo nephrectomy.	Moderate	Strong	100%
9b	What is the role for stenting of renal vascular injury or pelvi‐ureteric injury?	Urinary extravasation: In the presence of urinary extravasation, repeat imaging should be considered and a JJ stent or percutaneous drainage offered if increasing leak or in the presence of infection. Angiography with (selective) embolization should only be performed with clinical signs of persistent hematuria and contrast extravasation on CT. Stenting is not indicated for a devascularized kidney diagnosed on CT scan.	Low	Weak	90%
10a	Is there an optimal timing for relook after damage control surgery (DCS) chest and abdomen with open abdomen?	Timing of redo surgery: For re‐look surgery of the chest or abdomen, or for vascular temporary shunts, the most rapid return to the OR should be guided by organ injury and physiological restoration but ideally within 24–48 h.	Moderate	Weak	100%
10b	What about timing for definitive orthopedic surgery in stable versus unstable patients	Timing of orthopedic interventions: Early total care and repair of all injuries is potentially possible in the patient who has major injury without physiological compromise; however, for the patient who is defined as polytrauma delaying initial orthopedic intervention is advised and this should be guided by physiological status and may be augmented with the use of certain biomarkers to identify reversal of the inflammatory state.	Moderate	Weak	100%
11	Does the choice of skin preparation solution impact the incidence of wound sepsis in the trauma patient and if so, which is the preferred agent and carrier solution?	There is high quality evidence and current supporting guidelines that prefer alcohol‐based chlorhexidine solution. There is overall high‐quality evidence that chlorhexidine in alcohol is the skin preparation of choice. Povidone may be a suitable alternative if used in alcohol and not as the aqueous solution, particularly for orthopedic procedures.	Moderate	Strong	80%
12a	In trauma patients, particularly under specific conditions, does the administration of antibiotic prophylaxis reduce overall sepsis complications compared to no antibiotic use?	Antibiotic prophylaxis and duration: There is high quality evidence and numerous consensus guidelines that support perioperative prophylactic antibiotic therapy for maximally 24 h with appropriate redosing for extensive blood loss (4–6 units) and prolonged surgery (> 4 h).	High	Strong	100%
12b	What is the recommended duration of antibiotic prophylaxis in specific populations after trauma?	There is low evidence to extend antibiotic prophylaxis after 24 h in trauma and this should be considered on an individual basis and whether there is cavity contamination, giving short‐course therapy in preference to long‐course prophylaxis.	Low	Weak	100%
13	In trauma patients in the post‐operative period, what are the ICU sepsis screening tests of relevance and what benchmarks exist to guide therapy and quality of care compared to non‐trauma ICU patients?	There is moderate quality evidence to support routine application of surgical site infection and other sepsis guidelines as for other critically ill patients with the proviso that the inflammation of trauma must be considered as a potential confounding variable for a number of the sepsis markers. Clinical correlation with results of tests is highly supported and selective culture‐guided antibiotic use recommended.	Moderate	Strong	100%
14	In trauma patients with invasive lines what is the role of routine line‐change in prevention of sepsis compared to no routine change?	There is moderate quality evidence to support bundles for the insertion of central lines and urinary catheters and to support their removal on indication rather than routine line changes.	Moderate	Strong	100%
15	In cases requiring an esthesia for polytrauma and major trauma procedures what are the optimal balanced anesthesia options for best survival in this patient group?	Induction and maintenance of anesthesia and airway management carries a risk of cardiovascular collapse, especially in the hypovolemic patient. Fluid resuscitation should commence prior to induction of anesthesia in all hypotensive patients and those normotensive patients deemed to be significantly hypovolemic. Administration of anesthesia should be delayed until the operating theater or other point of hemorrhage control unless the patient's physiology mandates otherwise. Rational choices for administration of anesthesia are ketamine induction and maintenance, with consideration of low dose volatile maintenance of anesthesia.	Moderate	Strong	100%
16	In trauma patients with intra‐abdominal injury, what is the indication for the optimal type of drain device and optimal timing of removal of such drainage devices (including nasogastric tubes and urinary catheters)?	There is low quality evidence to support the removal of all drains and catheters at the earliest appropriate time guided by patient stability and clinical need. Drains are not indicated for the routine drainage of the peritoneal cavity or in the presence small or large‐bowel anastomosis.	Nasogastric tube: Low	Weak	100%
		In general drains may be removed at less than 30 mL/day for pancreatico‐biliary fistula.	Urinary drainage: Moderate	Weak	
			Abdominal drains: Low	Weak	
17	Does the type of hand‐scrub or the changing of gloves and instruments on closure of open surgical sites reduce the surgical site sepsis?	There is high quality evidence to support changing of gloves and using a new set of instruments for definitive sheath closure in abdominal surgery, extrapolation to other surgical procedures can be cautiously advocated.	Moderate	Weak	100%
18a	In trauma patients with an open abdomen do commercial systems versus home‐made TAC have a preferential role?	There is strong evidence that any TAC should include some form of negative pressure wound therapy, whether home‐made or commercial.	High	Strong	90%
18b	What is the role of mesh‐mediated traction versus skin only in delayed closure?	Abdominal reconstruction should be staged. There is moderate to strong evidence supporting staged abdominal reconstruction methods including dynamic fascial traction systems with negative pressure wound therapy in patients who tolerate it. Mesh mediated traction yields the highest primary fascial closure rates, and until a much later stage than the early TAC systems.	Moderate	Weak	90%
19a	What is the most appropriate choice of resuscitation fluids for trauma patients who require fluid resuscitation from the resuscitation phase to the post‐operative phase?	Trauma patients should be resuscitated according to a locally relevant massive transfusion protocol. This protocol should aim to restore, as near as possible, whole blood to the bleeding patient. As part of these protocols, plasma components should be administered as early as possible. Ideally these should be guided by dynamic coagulation assays such as thromboelstography if available. Crystalloid should be used as a last resort.	Moderate	Strong	100%
		Following hemostasis, initial hemoglobin targets of greater than 9 g/dL are rational, especially where the patient has an associated brain injury, or is at risk of ongoing/rebleeding, or requires further surgery. Lower transfusion thresholds (7 g/dL) may be considered in the subsequent phases of care.			
19b	What is the role for supplemental medications and procoagulant blood products in the trauma patient?	The use of tranexamic acid should be considered in different trauma systems depending on pre‐hospital transfer times and access to point of care coagulation tests or dynamic coagulation assays such as thromboelstography if available. Cryoprecipitate is not beneficial given empirically in the early phase, whereas fibrinogen and other factor concentrates may be administered based on tests of coagulation as close to the point of care as possible.	High	Strong	100%
20	What is the best management strategy for trauma patients in the operating theater—Does ARDSnet apply prior to ICU admission?	Evidence exists for optimal ventilator management of the trauma patient in the operating room. Best practices do include low to moderate tidal volume (6–8 mL/kg), moderate adjusted PEEP (5–8 cm H_2_O), and low driving pressures (< 15 cm H_2_O), although up to 10 mL/kg tidal volume may be considered acceptable in the operation room environment.	High	Strong	100%
21	What is the best ventilator strategy for trauma patients requiring mechanical ventilation in ICU?	Best practices are based on those patients undergoing major abdominal surgery. This includes low tidal volume (6–7 mL/kg), moderate PEEP, and low driving pressure (< 15 cm H_2_O).	Moderate	Strong	100%
22	Is there a role for ECMO in the management of trauma patients compared to no ECMO?	When available as a clinical option ECMO should be considered a viable option for severely injured trauma patients that require advanced mechanical ventilatory support.	Moderate	Strong	100%
23	What is the role of non‐invasive ventilation as a rescue therapy in the trauma patient versus invasive ventilation?	The use of non‐invasive ventilation as a primary or rescue therapy in trauma patients remains controversial and an option either as primary or a step‐down therapy. NIV appears to be a safe option for most patients and should not be reserved for those with high‐risk injury such as flail chest and a high thoracic injury score.	Low	Weak	100%
24	What is the best respiratory support for trauma patients with pulmonary contusion after initial assessment?	Trauma patients who sustain a pulmonary contusion should have judicious use of crystalloid to prevent worsening hypoxemia. General supportive measures utilizing both NIV and lung protective strategies may be implemented, while invasive ventilation is best for severe lung contusion meeting ARDSnet criteria.	Low	Weak	100%
25	What is the role for neuromuscular blockade agents in trauma patients in ICU versus nil?	NMBAs are used in the appropriate setting for trauma patients, but should be limited to bolus dosing for a maximum of 48 h duration. All patients should have BIS monitoring, if available, when paralyzed to ensure adequate depth of sedation.	Moderate	Weak	100%
26	In the trauma ICU patient who has an episode of aspiration, either during the resuscitation or later phases of care, what is the current best practice management and what is the role of bronchoscopy or antimicrobials in the management thereof?	Aspiration pneumonia is distinct from pneumonia caused by infection. There is moderate level evidence to perform early bronchoscopy, lavage and suction in these patients to allow differentiation and correct management. Antibiotics should not be routinely used until there is suspicion or evidence of infection.	Moderate	Strong	100%
27a1	In trauma patients in the ICU, what are the options for optimal analgesia and sedation to enhance recovery and early ICU mobility?	Multimodal analgesia‐focused analgo‐sedation incorporating non‐opioid non‐benzodiazepine drugs are recommended with sedation levels monitored using a validated tool.	Low	Weak	100%
27a2	Is ketamine a suitable combination with, or replacement for, propofol and opioids, or dexmedetomidine, for analgo‐sedation in trauma patients in ICU?	Ketamine is a safe and effective primary analgo‐sedation option, either with propofol or dexmedetomidine, or combined with rocuronium and fentanyl for intubation.	Low	Weak	100%
27a3	Is ketamine safe in polytrauma with TBI?	Ketamine is a safe option, even for TBI patients and does not increase intracranial pressure.	Low	Weak	100%
27a4	What additional alternative opioid‐sparing analgesia options exist for selected patients reduced ileus in trauma patients?	Dexmedetomidine and lignocaine infusions are newer safe adjuncts for opioid and benzodiazepine sparing in ICU, with advantages during weaning, which should be protocolized.	Low	Weak	100%
27a5	How should opioids be weaned in TICU to prevent dependence?	A weaning protocol is recommended.	Low	Weak	100%
27a6	What is the optimal analgosedation weaning protocol for planned extubation in ICU to avoid prolonged ventilation of trauma patients?	Drug substitution and spontaneous waking and breathing trials are recommended.	Very Low	Weak	90%
27b	In trauma patients in the ICU which drugs and substances are associated with withdrawal syndromes and what is the best options to prevent and treat these withdrawal episodes?	Preventing substance withdrawal is challenging with many confounding aspects and there are numerous options for treatment with clinical response to be assessed. Protocols improve outcome.	Moderate	Strong	90%
28	Thromboprophylaxis guidance	The authors quote and endorse the recently published trauma chapter of the European guidelines for thromboprophylaxis—see sub‐points below.	N/A	N/A	N/A
28a1	When should thromboprophylaxis in trauma patients commence and what type of prophylaxis is best in those without TBI?	There is strong evidence to support early initiation of thromboprophylaxis in patients with no brain injury: Thromboprophylaxis should be initiated early (< 24 h) after severe trauma without brain injury and in the absence of active hemorrhage.	High	Strong	100%
28a2	What is the role and timing of thromboprophylaxis in patients undergoing non‐operative management of high‐grade solid organ injuries?	For non‐operative management (NOM) of blunt solid organ injuries, VTE rates decrease consistently with early thromboprophylaxis but, based on conflicting results concerning delayed bleeding risk, some high‐risk patients might benefit from a 48‐h delay.	Low	Weak	90%
28a3	When should thromboprophylaxis in trauma patients commence and what type of prophylaxis is best in those with TBI?	In non‐operated patients with TBI and no progression of intracranial hemorrhage on the CT scan 24 h after the injury, we suggest early prophylaxis with LMWH within 48 h after injury.	Moderate	Weak	90%
28a4	When should thromboprophylaxis in trauma patients commence and what type of prophylaxis is best in those with TBI?	In patients having urgent neurosurgical interventions after TBI or in those at high risk of intracranial bleeding, we suggest delaying pharmacological prophylaxis on a case‐by‐case basis, balancing the risk of hemorrhage and the risk of VTE.	Moderate	Strong	100%
28a5	When should thromboprophylaxis in trauma patients commence and what type of prophylaxis is best in those with TBI?	For trauma patients with TBI and a contraindication to pharmacological prophylaxis, we recommend intermittent compression devices.	Moderate	Weak	90%
28a6	When should thromboprophylaxis in trauma patients commence and what type of prophylaxis is best in those with TBI?	We suggest adding low molecular weight heparins (LMWH) when the risk of bleeding decreases. In patients with spinal cord injury, we suggest starting pharmacological prophylaxis within 48 h following trauma or surgery.	Moderate	Strong	100%
28a7	When should thromboprophylaxis in trauma patients commence and what type of prophylaxis is best in those with spinal cord injury?	We suggest a total duration of pharmacological prophylaxis of 3–6 months after spinal cord injury with neurological deficit. We suggest combining pharmacological and IPC in patients with spinal cord injury and a motor deficit.	Low	Weak	100%
28b1	What type of prophylaxis is recommended?	We recommend that LMWH be used rather than UFH as thromboprophylaxis after severe trauma.	High	Strong	100%
28b2	What type of prophylaxis is recommended?	We suggest DOAC as an alternative to LMWH in protecting against VTE after acute care (weak evidence).	Low	Weak	90%
28b3	What type of prophylaxis dosing is recommended?	We suggest that dose adjustment of LMWH is associated with reduced VTE in severe trauma patients compared to standard dosing, but there is inconclusive evidence to support one method over another (i.e., weight adjusted vs. anti‐Xa levels) and further research is required.	Moderate	Weak	100%
28b4	In trauma patients in the ICU is there a role of screening for VTE risk?	We do not recommend the use of thromboelstography (TEG) or rotational thromboelastometry (ROTEM) to stratify VTE risk for adjusting prophylaxis.	Low	Strong	100%
28b5	What is the current role for IVC filters in severe trauma patients?	In trauma patients, we recommend against the routine use of IVC filters for the primary prevention of VTE.	Moderate	Strong	100%
28c1	In trauma patients in the ICU is there a role of ultrasound screening for VTE?	We conditionally recommend routine ultrasound DVT screening in high‐risk trauma patients in ICU as per local guidelines.	Low	Weak	100%
28c2	Is the potential benefit of withholding thromboprophylaxis prior to surgery outweighed by the risk of VTE complications?	There is also moderate evidence to question the rationale for withholding LMWH doses for interventions other than neurosurgery or spinal procedures. Existing limited evidence suggests that withholding thromboprophylaxis doses before surgery increases DVT rates.	Low	Strong	100%
29a	Does the timing (early vs. late) of RRT in trauma ICU patients impact on outcome?	Early initiation of renal replacement therapy in critically ill trauma patients does not confer improved survival. However, patients who undergo early RRT will have a shorter length of ICU and hospital stay, with a risk of more adverse events. Traditional indications for RRT are best followed but initiated early.	Moderate	Weak	90%
29b	What is the optimal type (CRRT vs. SLEDD vs. IHD) of RRT and current best practice indications for RRT in trauma ICU patients with AKI?	There is no clear evidence for RRT therapy that one mode is better than another, except for intermittent hemodialysis being poorly tolerated. An indication‐based approach is recommended, with liberal early initiation of RRT, earlier than traditional values. For crush/reperfusion syndrome there may be a place for early fluid restriction if anuric and early RRT. Mannitol and bicarbonate therapy are unnecessary and do not prevent the need for RRT.	High	Strong	100%
30a	What is the optimal timing of feeding in the critically ill trauma patient with continuous gut?	There is good quality evidence to support early enteral feeding using a gradual incremental protocol with naso‐enteric feeding reserved for those who have gastroparesis or feed intolerance, with the routine use of prokinetics and adjunctive treatments recommended. Following established nutrition society guidelines is supported for this goal. For planned surgery clear carbohydrate liquids till 2 h pre‐operative are recommended to avoid prolonged pre‐operative starvation in those tolerating enteral feed.	High	Strong	80%
30b	In trauma patients with discontinuous gut, what are the optimal feeding strategies and timing of feeding?	Commence enteral nutrition (oral or tube‐feed) within 6–8 h post‐surgery (trickle‐feed) once gut is continuous. Use parenteral nutrition by day 3–5 in those unable to achieve continuity of gut.	Moderate	Weak	100%
30c	To improve access to early enteral nutrition what therapies to prevent or treat post‐operative nausea and vomiting, or acute colonic ileus (‘Ogilvie‐type’) are best in trauma patients?	There is moderate quality evidence to support the prophylactic administration of multimodal therapies to prevent PONV and ileus and to identify and treat ileus or pseudo‐obstruction in ICU.	Moderate	Weak	100%
30d	In trauma patients who undergo planned operative intervention from the ICU or the general ward does carbohydrate loading improve outcome?	There is some evidence to support carbohydrate loading in the planned surgery patient improving outcomes with no adverse events.	Low	Weak	90%
30e	In patients after trauma who are intubated, does the feed need to be stopped more than 2 h prior to planned extubation?	Weak evidence exists for reduced feed‐stoppage duration prior to extubation exists and we recommend this decision be individualized per patient and in light of the underlying pathology and aspiration risk.	Low	Weak	100%
31	In trauma patients what is the role of optimal temperature management in those with coagulopathy, hypothermia or post traumatic cardiac arrest?	The overall aim of perioperative management is to achieve and maintain normothermia. If the patient has had a prolonged cardiac arrest/loss of cerebral perfusion targeted temperature management may be beneficial provided the patient's circulation is stable enough and bleeding is controlled. Hyperthermia (temp > 38°C) is known to be detrimental in brain injury.	Moderate	Strong	100%
32	In trauma patients, what are the indications and, are there contraindications, to early mobilization after trauma (except severe TBI and orthopedic restrictions) in ICU?	Trauma patients in the ICU who undergo a protocolized early mobilization program will have fewer days of mechanical ventilation. There is low quality evidence that early mobilization decreases the risk of delirium but is; however, associated with more adverse events in ICU.	Moderate	Strong	100%
33a	Ethics in trauma ICU: For trauma patients what is the best practice to provide advanced palliative care?	Early implementation of palliative care in patients with severe injury with an anticipated high risk of hospital mortality, permanent disability, or functional outcome incompatible with patient's wishes is advised. Prior health and pre‐injury functional status should be assessed. Standardized screening protocols and order sets increase compliance with timely palliative care intervention. We recommend an initial palliative assessment within 24 h of admission and a family meeting, if needed, within 72 h of admission to the ICU.	Moderate	Strong	90%
33b	What is the best practice to review and implement DNR orders in the trauma patient?	We recommend implementation of DNAR orders in the trauma patient, when appropriate, within the confines of local legal policies. This includes required validation or reconsideration, clear communication, and a timeline for DNAR re‐initiation if it is temporarily suspended.	High	Strong	100%
33c	In trauma patients with severe injury, what is the recommended approach to advanced care planning, whether for palliation or otherwise?	Implementation of advanced care planning on admission and within 72 h of hospitalization is recommended. This can facilitate concordance of patient care wishes, family engagement and appropriate patient focused care. Implementation results in a higher proportion of pre‐existing decisions being documented and reflected in future care of the trauma patient and avoidance of unwanted care.	Moderate	Strong	100%
34	In frail and elderly trauma patients, what is the best assessment and treatment approach to optimize outcome?	Trauma specific frailty screening scores are useful in advanced care planning. We cannot recommend one specific scoring system over another, but do recommend that an objective measure should be utilized. When possible, a geriatric trauma consultative service should be utilized to improve the best possible outcomes for elderly and frail patients.	Moderate	Strong	100%
35	In patients who survive major trauma or polytrauma what is the risk of PTSD and how can this be prevented and treated?	PTSD is common at 6–12 months post major trauma and treatment is a combination of psychotherapy, and pharmacotherapy adjusted to response. Prevention is difficult but screening is recommended.	Moderate	Strong	100%
36a	What is the evidence of benefit of trauma systems and does centralization of trauma offer advantages in outcomes?	Trauma systems do improve outcome but require the entire inclusive system to cooperate for the benefit of the patient and centralize the correct patent to the correct level of care.	Moderate	Strong	90%
36b	What is the status of development of ideal trauma systems versus real life in different countries, with different health systems, including in LMICs	Many systems are in the infant stage of development. Systems must be developed that are locally relevant, not simply copying HIC models as the only option. However, the generic elements are the same.	Moderate	Strong	90%
36c	What is the optimal time to transfer for rural and other trauma patients in non‐trauma hospitals to avoid under‐ or over‐triage? Does aeromedical transport offer benefits?	Triage to the correct level of care avoids overloading the system, whereas helicopter transfer has limited benefits in the urban setting but aeromedical support has value in the rural setting.	Moderate	Strong	90%
36d	Does police and public scoop and run improve outcomes as compared to waiting for emergency services?	For select penetrating injuries direct transfer avoiding EMS‐transfer may improve outcome.	Low	Weak	90%
36e	What systems of care should be offered for the older trauma patient with major trauma or polytrauma?	Geriatric trauma care requires multidisciplinary approaches with early goal of care planning.	Moderate	Strong	90%
37	What is the role of pediatric trauma centers versus pediatric trauma care in adult trauma centers? What is the ideal versus real life across the world?	There is no good evidence to support pediatric‐specific trauma centers outside a few high‐income countries and the local system must determine the most appropriate care for children.	Moderate	Weak	100%

*Note:* Percentage agreement.

**A. Acute phase decision‐making—pre‐operative**

1. Pre‐hospital care of trauma:

**PICO: In the major trauma and polytrauma patient, what are the pre‐hospital interventions that improve survival and reduce hospital length of stay compared to no intervention?**

Pre‐hospital trauma management is associated with reduced mortality when evidence‐based recommendations are followed. These include reducing the scene‐time, limiting pre‐hospital interventions in urban environments to life‐sustaining care, and control of bleeding [20, 21, 22]. The shortest possible prehospital time is associated with better survival in polytrauma and hemodynamically compromised patients. Limiting spinal motion restriction to indications and avoiding unnecessary collars and boards (especially in penetrating trauma) is a first step in ensuring optimal patient control [23, 24]. The use of tourniquets for arterial bleeding when the bleeding cannot be controlled with pressure or packing, limiting bolus crystalloid fluids and the use of hemostatic agents in wounds, along with tranexamic acid administration are steps that improve outcome [25, 26, 27, 28]. Junctional bleeding is best controlled by direct sustained pressure [29]. Advanced airway management should be limited to long‐transfers or aeromedical transfers [30]. Optimal airway management with mask ventilation alone versus endotracheal intubation is determined by the individual patient circumstances, recognizing that the goal is optimizing oxygenation and ventilation while avoiding adverse sequalae [31, 32]. Criteria for aeromedical evacuation after trauma have been recently refined [33]. Analgesia may be given using opioids or ketamine as drugs of choice, with ketamine, fentanyl, and rocuronium the drugs of choice for prehospital intubation, within the scope of practice locally [34, 35, 36, 37]. Additionally, the prehospital use of plasma or blood are currently under review, but not yet standard of care and no recommendation on this can be provided at present.

*1. Summary and recommendations:*

Rapid transfer to appropriate level of care with minimal fluid therapy or transfusions and attempts at bleeding control are the mainstay of prehospital trauma care. Tranexamic acid may be administered as per local policies. Advanced airway interventions should be restricted to selected patients and long‐distance transfers.

Level of evidence: Moderate

Grade of recommendation: Strong

**B. Trauma operative and non‐operative definitive care**

2. Surgical approach—operative decision‐making

**PICO: In polytrauma patients after initial assessment in the emergency department, what is the decision‐making philosophy the surgeon should follow to improve outcome?**

The surgical approach to a trauma patient is guided by the physiology primarily and secondarily by the organ injuries identified. Selective NOM (SNOM) is standard of care for hemodynamically normalized blunt injuries. For most penetrating trauma in the majority of centers operative intervention is the approach of choice, especially for those with physiological impairment with ‘damage control resuscitation and damage control surgery’ utilized as needed with definitive care more readily actioned with modern hemostatic resuscitation. In selected patients at selected centers with extensive penetrating trauma experience in correctly evaluated imaged patients, who are physiologically normal or improving, some evidence suggests NOM to be acceptable. For blunt trauma, especially those meeting polytrauma criteria consideration is given to initial use of ‘damage control’ philosophies and the use of various hemorrhage control adjuncts to temporize the patient while restoration of physiology is undertaken. Adjuncts may include temporary vascular shunting, other procoagulant products, or other adjuncts to prevent contamination or to control bleeding [38, 39, 40].

Most of the evidence for abdominal and chest damage control is from retrospective studies although the findings are similar in multiple centers across a number of countries, from the USA to South Africa. Orthopedic damage control in polytrauma patients is well established with better definition of the population and clinical guidelines in a recent major study [41]. Biomarkers to guide therapy include lactate and interleukin 6 [42, 43, 44, 45].

One main challenge remains the management of penetrating injuries to junctional areas where the ‘cavity of choice’ is less clear (e.g., thoraco‐abdominal—bleeding from both chest and into the abdomen and penetrating cervical trauma with bleeding also from the chest). Using FAST‐ultrasound screening, along with chest X‐ray, is a reliable method to choose the correct cavity [46, 47]. The other junctional area is the transition from abdomen/pelvis to the groins, since this may require either laparotomy or extra‐anatomical vascular access to control the bleeding, with the additional risk of limb ischemia complicating the clinical picture; however, this appears to be a less common issue in blunt trauma management [48, 49].

For this reason, the training of surgeons and anesthesiologists in trauma‐surgical decision‐making remains a mainstay of achieving better outcomes for trauma patients [50].

*2. Summary and recommendations:*

Clinical judgment is difficult to teach, but in trauma care this is an important aspect when deciding when to operate, when to be selective and how to perform the procedures, whether to go for definitive surgery (also called Early Total Care—ETC), or to temporize with damage control philosophies. It is recommended that the most experienced (trauma‐trained) surgeon should be the decision‐maker and perform the surgery.

Level of evidence: Moderate

Recommendation grade: Strong

3. Non‐Operative Management (NOM) versus Operative Management

**PICO: (3a1) In trauma patients who meet the current indications for NOM in solid organ injuries when is it still necessary to operate? (3a2) What about NOM in penetrating trauma? (3b) What is the role of repeat imaging after NOM for solid organ injuries in trauma patients? (3c) Does Early versus late feeding improve outcome in the trauma patient with solid organ injury? (3d) In solid organ injury managed by NOM, what is the role of bedrest compared to normal activity of daily living? (3e) In trauma patients who successfully undergo NOM of solid organ injury, what is the safe return to normal activity and sports activity timeline?**

The shift over the last decades from mainly operative to mainly non‐operative management (NOM) of solid organ injuries has been facilitated by access to high resolution CT scanning and improved resuscitation strategies. NOM in solid organ injuries is supported by a vast body of evidence [51, 52]. NOM involves employing strategies such as observation, embolization, and minimally invasive techniques to treat injuries to solid organs, including the liver, spleen, and kidneys without surgery, reducing the risks associated with invasive procedures while promoting faster recovery and preserving organ function. NOM is more widely applied in blunt trauma, whereas the most accepted approach to penetrating injuries to the abdominal cavity is still operative. In blunt trauma, the indication for surgery is dictated by deranged physiology, a lack of response to resuscitation and the identification of hollow viscus injuries requiring surgery [53].

In penetrating injuries (both stab and gunshot wounds), because of the high rate of non‐therapeutic explorative laparotomies, there is increasing support for NOM *in selected trauma centers* with extensive penetrating trauma experience, for hemodynamically normal(ized) patients with isolated injury to solid organs identified on CT scan, most notably the liver and kidneys [54, 55, 56]. However, this requires the patient to be awake, cooperative, with no signs of peritonitis, and there is a need for thorough clinical follow‐up, guided by imaging, hematoma severity, extravasation, clinical physiology, and initial response to resuscitation.

The evidence for routine repeat imaging for solid organ injuries has been consistent throughout the last 10–15 years, concluding that there is limited value in routine repeat imaging, in those with an uncomplicated hospital course [57, 58, 59]. Liver injuries and splenic injuries are either assessed together or separately. Indications for repeat imaging in liver injuries should be based only on clinical suspicion of complications. The same is valid for splenic injuries, except for the risk of pseudoaneurysm formation in high grade injuries that were embolized. Repeat imaging should hence be considered on day 3–5 in non‐embolized grade 3–5 splenic injuries [60]. CT scan should be replaced by contrast enhanced US if possible [61]. For renal trauma, repeat imaging should only be considered based on clinical signs and if urinary leak has been diagnosed on initial CT scan [58, 59]. In the pediatric population, there appears to be no indication for routine repeat imaging [62, 63, 64, 65].

There is no evidence to suggest early enteral feeding is contra‐indicated or has negative sequalae in NOM for solid organ injuries, provided that the patient condition allows for feeding. We suggest early enteral nutrition in all patients with bowel continuity [66, 67, 68]. Literature from 15 years ago already concluded that solid organ injuries do not require bedrest [69]. This was confirmed in more recent studies, both for the adult and the pediatric population [70, 71, 72].

The latest version of the American Pediatric Surgeons Association (APSA) guidelines (2018) confirmed support for the existing guidelines mandating avoiding contact sports and hard physical activity for grade of injury +2 in weeks (i.e., grade 4 = 6 weeks) for children [73]. For adults the evidence is less clear, but because there is insecurity in grading and adults heal less well, we conditionally suggest 8–12 weeks for grade 3–5 injuries, shorter for grade 1–2, and longer for anyone with persistent symptoms [74].

*3. Summary and recommendations:*

3a1. There is a high level of evidence to support NOM in hemodynamically normal patients with solid organ injuries after blunt trauma and no other indication for laparotomy provided the patient is appropriately monitored for deterioration.

Level of evidence: High

Recommendation grade: Strong

3a2. For penetrating injuries there is low level of evidence for the use of NOM in isolated liver or kidney injuries. SNOM in penetrating trauma is an option in selected experienced high‐volume centers with rapid access to operation facilities in case of SNOM failure.

Level of evidence: Low

Recommendation grade: Weak

3b. There is moderate to strong evidence for not repeating CT imaging for uncomplicated solid organ injuries. However, for grade 3–5 splenic injuries that have not been embolized we recommend contrast enhanced US or CT on day 3–5 to exclude pseudoaneurysm.

Level of evidence: Moderate to high

Recommendation grade: Strong

3c. We strongly recommend early enteral feeding for patients with NOM unless there is a specific contraindication because of the low incidence of harm.

Level of evidence: High

Recommendation grade: Strong

3d. Routine bedrest after NOM in solid organ injuries should be avoided and a limited period of activity restriction is recommended according to the patient age, type (grade) of injury and organ involved.

Level of evidence: Moderate

Recommendation grade: Strong

3e. Prolonged activity restrictions may be beneficial for contact sports and intense activity.

Level of evidence: Moderate

Recommendation grade: Strong

**C. Specific organ injuries**

4. Chest Wall Injury

4a. Chest wall Injuries and Rib Fixation

**PICO: (4a) What are the indications for operative rib fixation and the optimal timing in polytrauma and ICU‐requiring trauma patients with or without TBI?**

Surgical rib stabilization has experienced renewed interest over the past 5–10 years. There is, however, no level‐1 evidence determining which patients benefit from surgical stabilization of rib fractures (SSRF) or the optimal timing thereof. The highest level of evidence exists on ventilated patients with flail chest, in this subgroup SSRF is associated with 2.8 days more ventilator free days based on a medium size multicenter randomized controlled trial (RCT) from North American centers [75]. Additionally, one previous smaller single center RCT from Australia showed that SSRF of flail chest patients on the ICU was associated with 2 days shorter non‐invasive ventilation [76]. Neither of the studies showed mortality benefit among flail chest patients. RCTs so far have not shown benefit of SSRF for non‐ventilated patients with multiple rib fractures [77].

Several non‐randomized observational studies documented good outcomes of chest wall injured patients with SSRF in both ventilated and non‐ventilated patients with low complication rates. The generalization of these results is difficult because of the nature of the studies, the selection bias, and the lack of control population. There are however, two prospective studies of rib‐fixation in TBI polytrauma patients and both show some benefit [78, 79]. A recent large prospective observational cohort study on non‐operatively managed multiple rib fracture patients identified the contemporary benchmark for the management of multiple rib fractures as chest physiotherapy, pleural cavity drainage, and multimodal analgesic therapy, including timely regional blocks. This cohort of patients with a median of six rib fractures with mean age of 57 years, median ISS of 17, had an average 7 days of hospital stay, 2 days of ICU stay, 18% had pneumonia with very good 12‐month functional outcomes, and the mortality rate was 1.5% [80]. This was supported in three recent systematic reviews and meta‐analyses, reporting improved pneumonia incidence, slightly reduced length of stay, and minimal complications [81, 82, 83].

One RCT showed earlier fixation to have better outcomes than later (> 48 h) with some evidence of less inflammatory response in the early group and a shortened ventilator, ICU and hospital length of stay [84]. This same result was not found with the Dutch multicenter study, where there were no differences between fixation and non‐operative management [85, 86]. Late rib fixation for selected patients with persistent pain and non‐union is also safe and effective [87].

The focus of future interventional studies must achieve significantly better outcomes with SSRF than these contemporary outcomes with comprehensive non‐operative care. If it is decided to follow a SSRF management pathway, theoretically earlier surgical intervention is beneficial (compared to later surgery), but this is also the timeframe when multimodal pain therapy is likely to maximize its effect, thus further studies specifically focusing on timing of interventions are required to address this clinical question.

*4a. Summary and recommendations:*

There is moderate quality evidence to support rib‐fixation for flail chest or > 3 contiguous fractures, those with TBI and in the older patient, to reduce pneumonia, tracheostomy, ICU, and total hospital LOS. There is no evidence to prefer early versus later fixation, in selected patient populations.

Level of evidence: Moderate

Recommendation grade: Weak

4b. Chest wall injury: chest drain management

**PICO: (4b1) and (4b2) What are the indications for and the management of chest drains in the trauma patient and (4b3) at what point in the management is safe removal possible?**

The role of chest drains in the trauma patient are accepted practice as the treatment for hemothorax or pneumothorax, whether in the emergency department, the operating room, or in the ICU secondary often to procedural complications. Placement may be empiric in the resuscitation phase, after initial assessment and chest X‐ray, or even after CT‐scan, when the measured size of the pneumothorax is larger than 35 mm at the level of the third ribspace and in patients at risk of tension pneumothorax, such as due to positive pressure ventilation [88, 89, 90, 91]. What is controversial however, include the aspects of optimal chest‐tube size, type of reservoir, and timing of removal, and these questions are often challenged. The role of prophylactic antibiotics remains an area for debate with most studies not showing any clear role for pneumonia prevention, whereas some reduction in empyema in the penetrating cohort is recorded [92, 93]. Removal at end of expiration or maximal inspiration appears to make no difference [94, 95]. Additionally, how to manage excessive drainage and the role or lack thereof for drain‐suction therapy remain areas of debate. The latter will potentially delay mobility and as such is relevant to the ERATIC concept. The most recent review article (addressing both trauma and non‐trauma drains) suggests no difference with smaller versus larger tubes, and better outcome with short‐term suction overnight and removal on ‘waterseal’ at volumes varying from 50–200 mL/24 h, although a recent paper suggests this benefit of low‐pressure suction may be overstated [96, 97, 98, 99]. Addition of thoracic cavity suction evacuation prior to chest tube placement did not show a benefit in the one comparative cohort study; however, irrigation and suction appeared to reduce the incidence of retained hemothorax [100, 101]. Removal can be either performed in full inspiration or expiration with the ventilator, or with Valsalva during spontaneous breathing [94, 95, 102]. Modern valve‐based drains improve mobility and time to removal compared to traditional water‐seal devices and may enhance the opportunity for autotransfusion in patients in extremis [103, 104].

Suture closure or an occlusive dressing are equally effective. Conservative management of pneumothorax, even in those requiring general anesthesia (avoiding nitrous oxide), is now advocated in selected patients.

Mobility can be enhanced and removal facilitated by early physiotherapy, good analgesia, and exclusion of sub‐pulmonary hemothorax. This can result in tube removal in under 48 h in most simple traumatic hemothorax or pneumothorax as reported in sentinel papers from the 90s [105, 106, 107]. Removal while still on positive pressure ventilation appears safe, often around day 5–7 post ICU admission [108, 109].

Smaller chest tubes, including the use of pig‐tail catheters, appear to be as effective at drainage of pneumothorax and hemothorax with no increased risk of empyema and with smaller chest tubes providing better patient satisfaction. Removal using a Valsalva technique or a modified Valsalva technique is advocated [110, 111, 112, 113]. Complications can be of misplacement, unplanned removal, or technical issues and are mostly not in need of intervention [114, 115, 116, 117].

*4b. Summary and recommendations:*

4b1. Insertion of chest tube: There is strong evidence to support the therapeutic and prophylactic use of chest tubes for drainage of blood and air in the pleural space on an individualized basis.

Level of evidence: High

Recommendation grade: Strong

4b2.1. Size and position of insertion of chest tube: The optimal drain position is apico‐posterior. There appear to be no advantage of using larger tubes than 16Fr.

4b2.2. Complications are common and well detailed. The use of ultrasound can help guide the operator to place the tube appropriately.

4b2.3. Smaller tubes and pigtails along with valve‐based mobile drains reduce impaired mobility with equivalent drainage ability.

Level of evidence: Moderate

Recommendation grade: Strong

4c. Removal of chest tube: After appropriate clinical and sometimes radiological assessment the drains are often placed on low pressure suction and transitioned to water‐seal (off suction). Removal is considered when less than 200 mL of fluid drains in 24 h and there is no re‐accumulation of pneumothorax on ‘water‐seal’ in about 6 h. Drains can safely be removed, at either maximal inspiration or expiration, with the patient still in the ICU on positive‐pressure ventilation. Removal should include skin closure with either sutures or occlusive dressing or even cyanoacrylate glue. Routine post‐removal chest X‐ray is controversial.

Level of evidence: Moderate

Recommendation grade: Strong

5. Aortic Injury

**PICO: (5a) In trauma patients with minimal blunt aortic injury (BAI) is operative or non‐operative management required and what is the optimal management strategy compared to open surgery? (5b) For Moderate to severe aortic injury what is the optimal timing of repair: immediate versus delayed?**

Many BAI diagnosed with high resolution cross‐sectional imaging techniques have minimal aortic injury [118]. Up to 76% of grade I and II injuries can successfully be managed non‐operatively with medical therapy defined as heart rate control and afterload reduction [119]. In a large multicenter study, 40% of patients with BAI had minimal injuries, 60% of which were managed with medical therapy without aorta‐related complication or mortality [120]. These intimal injuries heal spontaneously and hence may be managed non‐operatively. However, the long‐term natural history of these injuries is not known, and hence caution should be exercised in using this form of treatment [119]. Target blood pressure and heart rates are < 100 bpm and < 100 mmHg SBP and management is guided by recent algorithms [121, 122, 123].

In a recent review of 2821 patients with blunt traumatic aortic injury, 75% underwent early (within 24 h of injury) thoracic endovascular aortic repair (TEVAR). Mortality was more than twofold in patients undergoing early TEVAR compared with delayed TEVAR (9.8% vs. 4.4%; *p* = 0.001). This mortality benefit persisted across injury severity groups and was independent of serious extra‐thoracic injuries. Patients undergoing delayed repair have improved survival compared with those repaired within the first 24 h of injury in spite of similar injury patterns and severity [124].

*5. Summary and recommendations:*

5a. There is moderate quality evidence to recommend non‐operative management with medical therapy for minimal aortic injuries, defined as grade I or II by the Society of Vascular Surgery Grading Scale. Serial cross‐sectional imaging is recommended to assure radiographic resolution of injury.

Level of evidence: Moderate

Recommendation grade: Strong

5b1. There is weak quality evidence to recommend that patients with BAI should undergo TEVAR at, or greater than, 24 h (compared to under 24 h) to improve overall survival.

5b2. Open surgery is restricted to a limited subset of patients in extremis.

Level of evidence: Low

Recommendation grade: Weak

6. Splenic Injury

**PICO: For major splenic injury, is main artery versus selective embolization (SAE) better and is it safer to embolize versus observe in high grade injuries? How effective is immune function after SAE for major splenic injury?**

As an important management concept, grossly unstable patients with intraabdominal bleeding require surgery and if the spleen is injured, a splenectomy should be performed. There is strong evidence that for the adult patient who is stable or has normalized upon resuscitation, a CT scan with ongoing extravasation from a splenic injury should undergo SAE [125]. There is also moderate and increasing evidence to support preemptive SAE for hemodynamically normal(ized) OIS grade 4 and 5 splenic injuries, whereas stable OIS grade 1 and 2 should be observed [126]. There is moderate evidence suggesting that splenic OIS grade 3–5 injuries treated nonoperatively without SAE should be investigated by contrast enhanced ultrasound, or CT scan on day 3–5 [127]. Whether to perform central or selective embolization or both remains controversial, but non‐selective appears to be the more common option [128, 129].

Existing moderate evidence suggests that an embolized spleen retains immune function, and therefore conditionally suggest that these patients should not be immunized [130, 131].

*6. Summary and recommendations:*

6a. Spleen injury can be managed non‐operatively for low grade injury and with addition of AE for higher grade injury. Where to embolize anatomically is controversial, but we conditionally recommend non‐selective SAE.

6b. Immunity appears to be retained after SAE.

Level of evidence: Moderate

Grade of recommendation: Strong

7. Liver Injury

**PICO: (7a) Which trauma patients benefit from liver packing versus resection? (7b) For liver injury when is it best to embolize versus observe? (7c) What is the role of ERCP in the management of hepatic trauma and the optimal timing of drainage for liver trauma related biliary leakage?**

There has been a dramatic shift in the treatment of blunt liver injuries over the past 20 years; from mainly operative to mostly NOM with or without AE. Improved resuscitation strategies have allowed patients to stop bleeding and increase the NOM rates [132]. From performing angiography in most severe liver injuries and for every contrast blush, there is increasing support for reserving angiography for the patients with clinical signs of ongoing bleeding and to clinically observe the hemodynamically normal(ized) patients [133].

The hemodynamically grossly compromised patient who does not respond to resuscitation still needs an operation (30% of grade 4–5 injuries), and mortality in this group remains high at 40%–50% [39, 134, 135]. With damage control resuscitation strategies, these liver injuries can mostly be managed with systematic packing and temporary abdominal closure (TAC) until normalized physiology. The need for subsequent AE is to be assessed on an individual basis, complications should be expected and treated when necessary [136]. Closed suction drainage is indicated after unpacking, for bile leak [137]. ERCP is invasive with a risk of complications, is seldom indicated but should be considered early when CBD injury is suspected, and later if persistent high output bile leak (many weeks), as well as in the presence of later bile duct obstruction to achieve downstream control [138, 139, 140].

*7. Summary and recommendations:*

7a. The hemodynamically compromised patient who does not respond to resuscitation still needs an operation which should be urgently undertaken. With damage control resuscitation strategies, these liver injuries can mostly be managed with systematic packing and TAC until normalized physiology.

Level of evidence: Moderate

Recommendation grade: Strong

7b. Patients with blunt liver injury and who are hemodynamically stable after resuscitation should undergo NOM. Angiography should be reserved for patients with clinical signs of ongoing bleeding (not only persistent CT‐contrast extravasation or hemobilia).

Level of evidence: Moderate

Recommendation grade: Strong

7c. For patients with suspected bile duct injury there is moderate evidence supporting closed suction drainage for bile leak. ERCP should be reserved for patients with suspicion of CBD injury and if persistent high output bile leak after several weeks.

Level of evidence for closed suction drainage: Moderate

Level of evidence for ERCP: Low

Recommendation grade: Weak

8. Pancreatic Injury

**PICO: (8a) When should a patient with pancreas injury undergo observation versus operative management? (8b) When is distal pancreatectomy the procedure of choice? (8c) What is the optimal management of grade 4 and 5 injury?**

The literature shows that the treatment of pancreatic injuries (PI) varies widely, and there is lack of prospective and well controlled retrospective studies. PI are associated with high morbidity rates. However, there is support for NOM of grade 1 and 2 (no main duct injury) PIs [141]. For OIS grade 3 injuries (duct injury to the left of SMA) practice and reported results tend to report distal pancreatectomy, while in the pediatric population it is divided between resection (spleen preserving) and observation [142, 143]. For grade 4 and 5 (pancreatic duct injury to the right of SMA), initial wide drainage is recommended, and Whipple's procedure is reserved for massive destruction involving CBD and duodenum and the reconstruction should be performed after physiology has been restored in ICU [144]. CT is the gold standard for radiologic diagnosis but with limited sensitivity. However, sensitivity for CBD injury might be as low as 37% for MRCP [145]. Fistula rate post pancreatectomy is significant, but ERCP is very seldom indicated, as these fistulae heal if given time, mostly within 14 days but can be up to 10 weeks [146].

*8. Summary and recommendations:*

8a. Grade 1 and 2 Pancreatic injuries (no ductal injury) should initially be treated non‐operatively.

Level of evidence: Moderate

Recommendation grade: Strong

8b. Grade 3 injuries (to the left of the superior mesenteric artery) can be treated with distal pancreatectomy but there remains the risk of fistula. For those managed with NOM there is a risk of sepsis and pseudocyst.

Level of evidence: High

Recommendation grade: Strong

8c. Grade 4 and 5 injuries should initially be treated with wide drainage rather than primary resection with subsequent intervention planning as indicated.

Level of evidence: Moderate

Recommendation grade: Weak

9. Kidney Injury

**PICO: (9a) In the patient with high grade renal injury—what is the role of NOM with or without angiography? What is the role of NOM versus nephrectomy? (9b) What is the role for stenting of renal vascular injury or pelvi‐ureteric injury?**

The literature on renal injuries (RI) is consistent over the last decades but mainly consists of retrospective studies. There is wide agreement for NOM of OIS grades 1 and 2 injuries. For high grade (OIS grade 3–5) renal injuries, the success rate depends on injury grade, hemodynamic stability and transfusion requirements as well as the presence of urinary extravasation. With improved resuscitation strategies more patients stabilize and lend themselves to NOM. However, the patients with high grade RI who require an operation are most likely to undergo nephrectomy [147]. In the presence of urinary extravasation, repeat imaging should be considered and a JJ stent or percutaneous drainage offered if increasing leak or in the presence of infection.

Angiography and embolization are associated with severe complications and should be performed only when clinically indicated: in the presence of clinical signs of ongoing bleeding and contrast extravasation on CT scan, a delayed presentation pseudoaneurysm, or traumatic arterio‐venous malformation. A devascularized kidney, when diagnosed on CT, is unlikely to benefit from stenting as warm ischemia time will always be too long [148, 149].

In the pediatric population, there is wide consensus for NOM of all grades of RI when physiology allows [150, 151].

*9. Summary and recommendations:*

9a. Renal injury: There is strong evidence supporting NOM in all grade RI in hemodynamically normal or normalized patients (including children) with no other indication for laparotomy. The patients with high grade RI who require an operation will likely undergo nephrectomy.

Level of evidence: Moderate

Recommendation grade: Strong

9b. Urinary extravasation: In the presence of urinary extravasation, repeat imaging should be considered and a JJ stent or percutaneous drainage offered if increasing leak or in the presence of infection.

Angiography with embolization should only be performed with clinical signs of persistent hematuria and contrast extravasation on CT. Arterial stenting is not indicated for a devascularized kidney diagnosed on CT scan.

Level of evidence: Low

Recommendation grade: Weak

**D. Early intraoperative and post‐operative aspects**

10. Timing of relook surgery in damage control.

**PICO: (10a) Is there an optimal timing for relook after damage control surgery (DCS) chest and abdomen with an open abdomen? (10b) What about timing for definitive orthopedic surgery in stable versus unstable patients?**

DCS and temporary abdominal closure (TAC) has for almost three decades been the main strategy in patients who are hemodynamically compromised after trauma, requiring massive transfusions, and requiring operative management for abdominal injuries and bleeding. Literature in this area is of limited quality and mainly consists of retrospective cohort descriptions. Improved resuscitation strategies have reduced the need for laparotomies and DCS. However, the indications remain the same even if fewer qualify. Existing and more recent evidence is not able to define a magic timeline for relook surgery but rather underlines the fact that physiology determines timing, and that the earlier definitive closure can be achieved the better.

The patient, injury pattern, and type of resuscitation affects this decision. We would suggest to aim for 24–48 h for torso reoperations [152] Multidisciplinary teams and surgical involvement in the ICU phase is of vital importance [153]. When multiple interventions are indicated, consensus statements support multidisciplinary assessments, where physiology helps avoid overzealous early surgery, at the same time prioritizing definitive surgery in a sequence that minimizes risk of complications [13, 154, 155]. For orthopedic damage control procedures, the optimal timing of re‐intervention can be guided by various physiological biomarkers which in various studies have included interleukin 6 levels, lactate clearance, and by excluding occult hypoperfusion. On average this appears to be somewhere between day 5 and 7 post‐injury, especially where either TBI or major chest injury is present in the polytrauma patient [41, 42, 43, 154, 155, 156].

*10. Summary and recommendations:*

10a. Timing of Surgery:

For re‐look surgery of the chest or abdomen, or for vascular temporary shunts, the most rapid return to the OR should be guided by organ injury and physiological restoration but ideally within 24–48 h.

Level of evidence: Moderate

Recommendation grade: Weak

10b. Timing of Orthopedic interventions:

Early total care and repair of all injuries is potentially possible *in the patient who has major injury without physiological compromise*; however, for the patient who is defined as polytrauma delaying initial orthopedic intervention is advised and this should be guided by physiological status and may be augmented with the use of certain biomarkers to identify reversal of the inflammatory state.

Level of evidence: Moderate

Recommendation grade: Weak

11. Skin asepsis

**PICO: Does the choice of skin preparation solution impact the incidence of wound sepsis in the trauma patient and if so, which is the preferred agent and carrier solution?**

Surgical site infection (SSI) is common after trauma surgery [157]. The method of skin preparation for trauma surgery has remained an area of discussion, with alcohol‐based solutions holding promise as the more reliable solution, provided the alcohol is allowed to evaporate. Many surgeons prefer an iodophore solution as they claim it shows the surface area that had been cleaned. There is no overt consensus; however, all surgeons desire to reduce SSI with skin preparation one aspect thereof [158]. Reviewing the literature shows that for trauma most of the experience is in the orthopedic trauma arena, with the results suggesting that there is no difference if using the aqueous solutions, but reduced rate of SSI when using alcohol‐based chlorhexidine solution compared with an iodophore [159, 160, 161, 162, 163]. This was also supported in a study on elective abdominal surgery and was confirmed in four recent meta‐analyses, whereas a recent clinical study of chlorhexidine showed no reduction in bacterial killing ability over time even with prolonged use [164, 165, 166, 167, 168]. Some recent studies, outside the trauma environment, show that dual or sequential use of both agents may have some benefit in reducing bacterial colonies but this did not translate into reduction in SSI. The benefit may be due to the use of alcohol‐based solutions *rather than the actual antiseptic agent* in the solution [169, 170, 171, 172, 173]. The important confounder is that most evidence is from, either orthopedic, and clean or clean‐contaminated surgery making the recommendation an extrapolation for trauma and chlorhexidine in alcohol as the preferred agent for non‐orthopedic procedures [174, 175, 176, 177].

*11. Summary and recommendations:*

There is high quality evidence and current supporting guidelines that prefer alcohol‐based chlorhexidine solution. There is overall high‐quality evidence that chlorhexidine in alcohol is the skin preparation of choice. Povidone may be a suitable alternative if used in alcohol and not as the aqueous solution, particularly for orthopedic procedures.

Level of evidence: Moderate

Recommendation grade: Strong

12. Antibiotic prophylaxis and duration

**PICO: (12a) In trauma patients, particularly under specific conditions, does the administration of antibiotic prophylaxis reduce overall sepsis complications compared to no antibiotic use? (12b) What is the recommended duration of antibiotic prophylaxis in specific populations after trauma?**

Trauma patients constitute a high‐risk population prone to infection, as trauma and interventions can disrupt the body's natural barriers, leading to contamination. Antibiotic prophylaxis (AP) is a commonly used intervention in trauma care aimed at reducing infection rates [178, 179] However, after decades of liberal antibiotic use, the emergence of drug‐resistant bacteria pose an increasing risk. The use of AP should adhere to current available evidence to maximize its benefits while minimizing the risk of misuse. Two distinct trauma‐related conditions are included: meningitis in severe skull fractures, and central nervous system (CNS) infection in external ventricular drains (EVD) or intra‐cranial pressure (ICP) monitors.

An updated Cochrane systematic review and meta‐analysis on AP for preventing meningitis in basilar skull fractures was published in 2015 [180]. The review did not identify any new randomized controlled trials (RCTs) compared to the previous review in 2011 by the same group but it included five RCTs for analysis. Additionally, they identified 17 non‐RCTs for analysis. The review does not support the use of AP in patients with basilar fractures, regardless of the presence of cerebrospinal fluid leakage. Furthermore, the results from non‐RCTs were consistent with those from the randomized data.

A single‐center case series with a systematic review, published in 2023, evaluated AP for penetrating traumatic brain injury. Although the literature review did not reveal any significant benefit of AP, all patients who did not receive AP developed CNS infection in their case series. Consequently, the authors proposed a short course of *therapeutic* antibiotics for patients with penetrating traumatic brain injury [181].

An evidence‐based consensus statement from the Neurocritical Care Society conditionally recommends one dose of antimicrobials prior to EVD insertion and strongly recommends against the continuous use of antimicrobials during placement [182]. However, a recent retrospective cohort study did not demonstrate the benefit of AP in patients with EVDs or ICP monitors [183]. Given that the quality of the studies published to date on AP for skull fractures and EVD or ICP monitor insertion is insufficient, the effectiveness of AP in these populations cannot be determined and requires appropriately designed RCTs.

For other truncal trauma the recommendations follow that of clean contaminated surgery, namely that in general, no prophylaxis should be given to blunt trauma patients unless a hollow viscus injury is suspected or identified. Antibiotic prophylaxis for penetrating trauma should not last more than 24 h in the absence of hollow viscus injuries and broad‐spectrum antibiotics with aerobic and anaerobic bacteria coverage are preferred while aminoglycosides should be avoided. In the case of hemorrhagic shock and associated acute kidney injury, the dose of antibiotics should be adjusted with a second dose if more than 4–6 units of blood are required [184]. With abdominal pathology, the presence of contamination, underlying physiology, and core temperature also impact the risk of SSI. For limb trauma, open fractures benefit from prophylaxis for no more than 24 h while prolonged prophylaxis is traditionally used in maxillo‐facial trauma. Tetanus toxoid where appropriate and antiviral vaccines (e.g., rabies) should be used prophylactically if indicated. Where antibiotic therapy is given shortened (4–5 days) courses are preferred over longer (7–10 days) courses and are terminated early [184, 185, 186].

*12. Summary and recommendations:*

Antibiotic prophylaxis and duration: There is high quality evidence and numerous consensus guidelines to support perioperative prophylactic antibiotic therapy for maximally 24 h with appropriate re‐dosing for extensive blood loss (4–6 units) and prolonged surgery (> 4 h). There is low evidence to extend antibiotic prophylaxis after 24 h in trauma and this should be considered on an individual basis and whether there is cavity contamination, giving short‐course therapy in preference to long‐course prophylaxis.

12a. 24‐h maximal prophylaxis

Level of evidence: High

Recommendation grade: Strong

12b. Extension of Prophylaxis beyond 24 h: if needed use therapeutic course

Level of evidence: Moderate

Recommendation grade: Strong

## 4 Aspects From Part 2 Relevant to Part 1

The following five PICO questions are detailed in part 2, but aspects thereof are relevant to initial management and are duplicated here in the form of a brief summary, with the relevant recommendation: The reader is referred to part 2 for the detailed literature discussion and related references.

15. Anesthesia

**PICO: In cases requiring anesthesia for polytrauma and major trauma procedures what are the optimal balanced anesthesia options for best survival in this patient group?**

Induction and maintenance of anesthesia and airway management carries a risk of cardiovascular collapse, especially in the hypovolemic patient. Fluid resuscitation should commence prior to induction of anesthesia in all hypotensive patients and those normotensive patients deemed to be significantly hypovolemic. Administration of anesthesia should be delayed until the operating theater or other point of hemorrhage control unless the patient's physiology mandates otherwise. Rational choices for administration of anesthesia are ketamine induction and maintenance outside the operating room with consideration of low dose volatile maintenance of anesthesia.

Level of evidence: Moderate

Recommendation grade: Strong

17. Abdominal Closure tray and glove changes

**PICO: Does the type of hand‐scrub or the changing of gloves and instruments on closure of open surgical sites reduce the surgical site sepsis?**

There is high quality evidence to support changing of gloves and using a new set of instruments for definitive sheath closure in abdominal surgery, extrapolation to other surgical procedures can be cautiously advocated.

Level of evidence: Moderate

Recommendation grade: Weak

19a. Resuscitation/use of blood products and coagulation management

**PICO: What is the most appropriate choice of resuscitation fluids for trauma patients who require fluid resuscitation from the resuscitation phase to the post‐operative phase?**

Trauma patients should be resuscitated according to a locally relevant massive hemorrhage protocol. This protocol should aim to restore, as near as possible, whole blood to the bleeding patient. As part of these protocols, plasma components should be administered as early as possible. Ideally these should be guided by dynamic coagulation assays such as thromboelstography if available. Crystalloid should be used as a last resort.

Level of evidence: Moderate

Level of recommendation: Strong

**PICO: (19b) What is the role for supplemental medications and procoagulant blood products in the trauma patient?**

The use of tranexamic acid should be considered in different trauma systems depending on pre‐hospital transfer times and access to point of care coagulation tests or dynamic coagulation assays such as thromboelstography if available. Cryoprecipitate is not beneficial given empirically in the early phase, whereas fibrinogen and other factor concentrates may be administered based on tests of coagulation as close to the point of care as possible.

Level of evidence: High

Level of recommendation: Strong

**PICO: What is the best ventilation management strategy for trauma patients in the Operating Room—Does ARDSnet apply prior to ICU admission?**

Evidence exists for optimal ventilator management of the trauma patient in the operating room. Best practices do include low to moderate tidal volume (6–8 mL/kg), moderate adjusted PEEP (5–8 cm H_2_O), and low driving pressures (< 15 cm H_2_O), although up to 10 mL/kg tidal volume may be considered acceptable in the operation room environment.

Level of evidence: High

Grade of recommendation: Strong

## 5 Conclusion

This paper has detailed the initial assessment and management of the polytrauma and major trauma patient from the perspective of prehospital, initial emergency care (operative and non‐operative), and immediate organ injury management aspects, aiming to optimize physiological status and reduce complications, thus reducing the length of hospital stay. The following two papers will address post‐operative intensive care and systems aspects respectively.

## Author Contributions

**Timothy C. Hardcastle:** conceptualization, methodology, data curation, resources, project administration, writing – review and editing, writing – original draft, investigation, validation, supervision. **Christine Gaarder:** conceptualization, methodology, data curation, writing – review and editing, project administration, investigation, supervision. **Zsolt Balogh:** data curation, investigation, writing – review and editing, formal analysis. **Scott D'amours:** data curation, validation, investigation, formal analysis, writing – review and editing. **Kimberly A. Davis:** data curation, validation, investigation, writing – review and editing. **Amit Gupta:** data curation, investigation, writing – review and editing. **Shahin Mohseni:** methodology, data curation, investigation, validation, formal analysis, writing – review and editing. **Paal A. Naess:** data curation, investigation, writing – original draft, writing – review and editing, formal analysis. **Shanisa Naidoo:** data curation, investigation, formal analysis, writing – review and editing. **Tarek Razek:** investigation, writing – review and editing, formal analysis, data curation, resources. **Simon Robertson:** writing – review and editing, investigation, formal analysis. **Hayaki Uchino:** investigation, formal analysis, writing – review and editing, data curation. **David Zonies:** data curation, investigation, formal analysis, writing – review and editing. **Jade Whing:** investigation, formal analysis, validation, data curation, project administration, writing – review and editing. **Michael J. Scott:** methodology, data curation, supervision, writing – review and editing, writing – original draft, project administration, investigation.

## Conflicts of Interest

Timothy C. Hardcastle, Christine Gaarder, Zsolt Balogh, Scott D'amours, Kimberly A. Davis, Amit Gupta, Shahin Mohseni, Paal A. Naess, Shanisa Naidoo, Tarek Razek, Simon Robertson, Hayaki Uchino, David Zonies, and Jade Whing have no conflicts of interest. Michael J. Scott, representing the ERAS group, has honoraria from and serves on advisory boards of Baxter, Edwards Lifesciences, Deltex, Trevena, and Merck. He also receives travel reimbursement from these companies and is Past President of ERAS USA.

## Supporting information

Supporting Information S1

Supporting Information S2

## Data Availability

The data that support the findings of this study are available on request from the corresponding author. The data are not publicly available because of privacy or ethical restrictions.
