# Supplementary figures and images for: Guidelines for Enhanced Recovery After Trauma and Intensive Care (ERATIC): Enhanced Recovery After Surgery (ERAS) Society and International Association of Trauma Surgery and Intensive Care (IATSIC) Recommendations: Paper 1: Initial Care—Pre and Intraoperative Care Until ICU, Including Non‐Operative Management
Source: World J Surg. 2025 Jul 22;49(8):1997–2028. doi: 10.1002/wjs.70002 (PMC12338446; doi:10.1002/wjs.70002)

**Supplementary Material**

**Addendum 1: Search process flow-chart (generic) ERATIC Process**


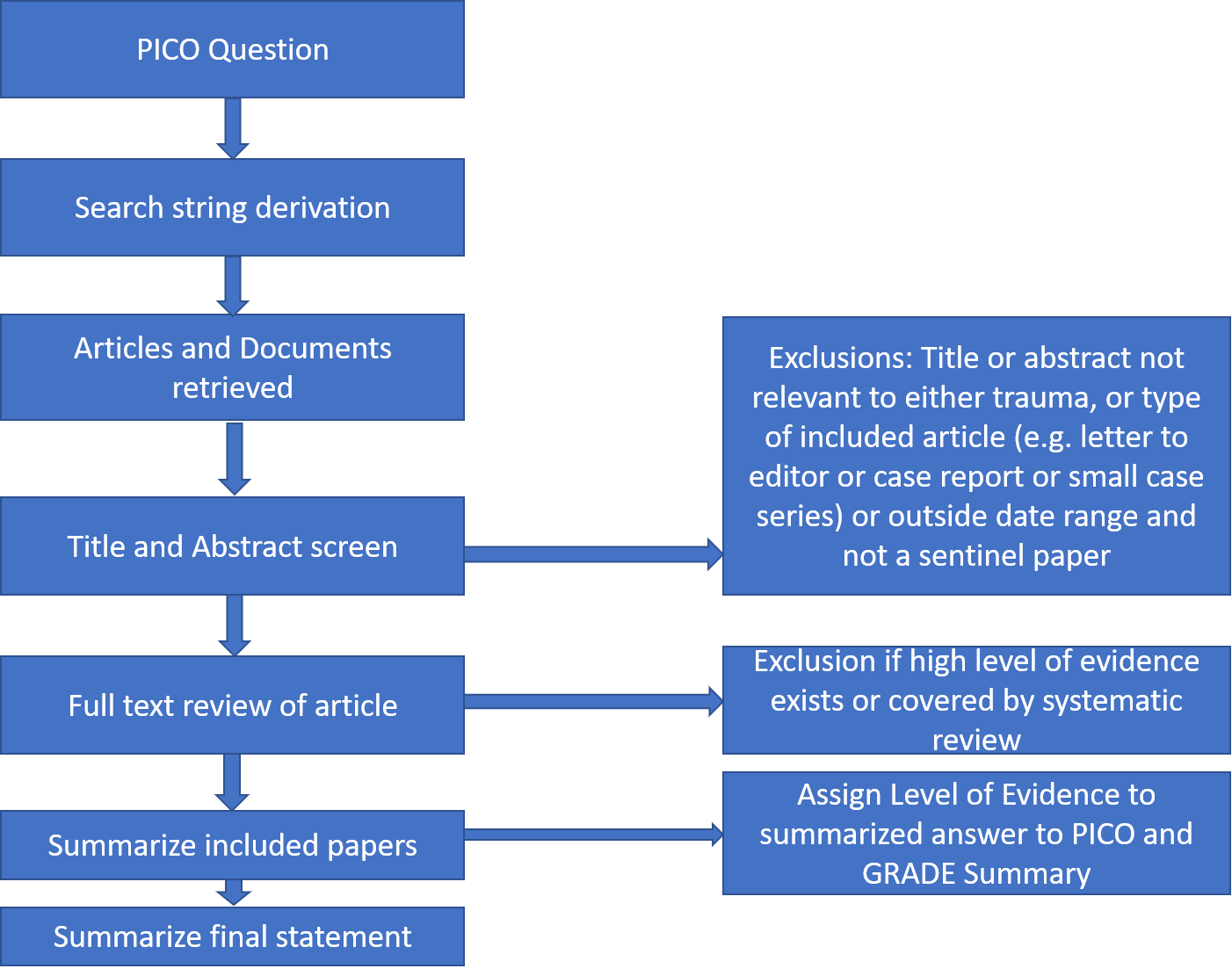

Supplement: Supplementary file 1 — Supporting Information S1 [file WJS-49-1997-s002.docx]
