# Supplementary material for: Guidelines for Enhanced Recovery After Trauma and Intensive Care (ERATIC): Enhanced Recovery After Surgery (ERAS) Society and International Association of Trauma Surgery and Intensive Care (IATSIC) Recommendations: Paper 1: Initial Care—Pre and Intraoperative Care Until ICU, Including Non‐Operative Management
Source: World J Surg. 2025 Jul 22;49(8):1997–2028. doi: 10.1002/wjs.70002 (PMC12338446; doi:10.1002/wjs.70002)
Supplement: Supplementary file 2 — Supporting Information S2 [file WJS-49-1997-s001.docx]

**Addendum 2**

**Examples of search-terms and search strings for PICO questions. This is illustrative of all the searches and is not an exhaustive list.**

**Example 1**

**“Does early bronchoscopy assist in diagnosis and management of aspiration pneumonia in trauma patients?”**

"Bronchoscopy"[Mesh] OR Bronchoscop*[tiab] MeSH: "Bronchoscopy"[Mesh]

AND

"Pneumonia, Aspiration"[Mesh] OR Aspiration*[tiab] OR “Aspiration pneumonia*”[tiab]
BUT NOT "Foreign Bodies"[Mesh] OR “Foreign body”[tiab] OR “Foreign bodies”[tiab] OR Inhalation[tiab]

AND

"Wounds and Injuries"[Mesh] OR Trauma[tiab] OR “Trauma patient*”[tiab] OR Injured[tiab] OR “Traumatic injur*”[tiab] OR Injur*[tiab]

AND

"Hospitalization"[Mesh] OR Hospitaliz*[tiab] OR Hospitalis*[tiab] OR “Hospitalized patient*”[tiab]

AND

MeSH: "Intensive Care Units"[Mesh]

**Example 2**

**“Sepsis screening and risk for HAP/VAP/SSI in trauma ICU”**

"Sepsis"[Mesh] OR sepsis[tiab] OR screen*[tiab] OR detection* OR “early detection”[tiab] OR diagnos*[tiab]

AND

"Cross Infection"[Mesh] OR **“**Healthcare associated infection*”[tiab] OR “hospital acquired infection*”[tiab] OR “nosocomial infection*”[tiab]

AND
"healthcare associated pneumonia"[Mesh] OR "Pneumonia, Ventilator-Associated"[Mesh] OR **“**ventilator associated pneumonia*”[tiab]

OR

"Surgical Wound Infection"[Mesh] OR “surgical site infection*”[tiab] OR “surgical wound infection*”[tiab]

OR

"Anti-Bacterial Agents"[Mesh] OR "Antibiotic Prophylaxis"[Mesh] OR **“**anti infective agent*”[tiab] OR antimicrobial*[tiab] OR “anti-bacterial agent*”[tiab] OR antibiotic*[Tiab]

AND

"Wounds and Injuries"[Mesh] OR Trauma[tiab] OR “Trauma patient*”[tiab] OR Injured[tiab]

AND

"Hospitalization"[Mesh] OR Hospitaliz*[tiab] OR Hospitalis*[tiab] OR “Hospitalized patient*”[tiab]

OR
"Intensive Care Units"[Mesh] OR "Critical Care"[Mesh] OR “intensive care unit*”[tiab] OR ICU[tiab] OR “critical care”[tiab]

**Example 3**

**“Is chlorhexidine more effective than other options as skin antisepsis for reducing surgical site infection?”**

"Antisepsis"[Mesh] OR "Skin"[Mesh] OR **“**skin antiseps*”[tiab] OR “skin aseps*”[tiab] OR disinfect*[tiab] OR steriliz*[tiab] OR "Antisepsis"[Mesh] OR "Skin"[Mesh] OR **“**skin antiseps*”[tiab] OR “skin aseps*”[tiab] OR “skin preparation*”[tiab]

AND

"Chlorhexidine"[Mesh] OR "Disinfectants"[Mesh] OR chlorhexidine*[tiab]

"Chlorhexidine"[Mesh] OR chlorhexidine*[tiab]

AND

"Wounds and Injuries"[Mesh] OR "Traumatology"[Mesh] OR Trauma[tiab] OR “Trauma patient*”[tiab] OR Injured[tiab] OR “Traumatic injur*”[tiab] OR Injur*[tiab]

AND

"Emergency Treatment"[Mesh] OR "General Surgery"[Mesh] OR **“**emergency surger*”[tiab] OR “urgent surger*”[tiab]

AND

"Surgical Wound Infection"[Mesh] OR “surgical site infection*”[tiab] OR “surgical wound infection*”[tiab]
